# Supplementary material for: MtDNA Analysis Indicates Human-Induced Temporal Changes of Serbian Honey Bees Diversity
Source: Insects. 2021 Aug 27;12(9):767. doi: 10.3390/insects12090767 (PMC8472511; doi:10.3390/insects12090767)
Supplement: Supplementary file 1 [file insects-12-00767-s001.zip › Figure S1-S5.pdf]

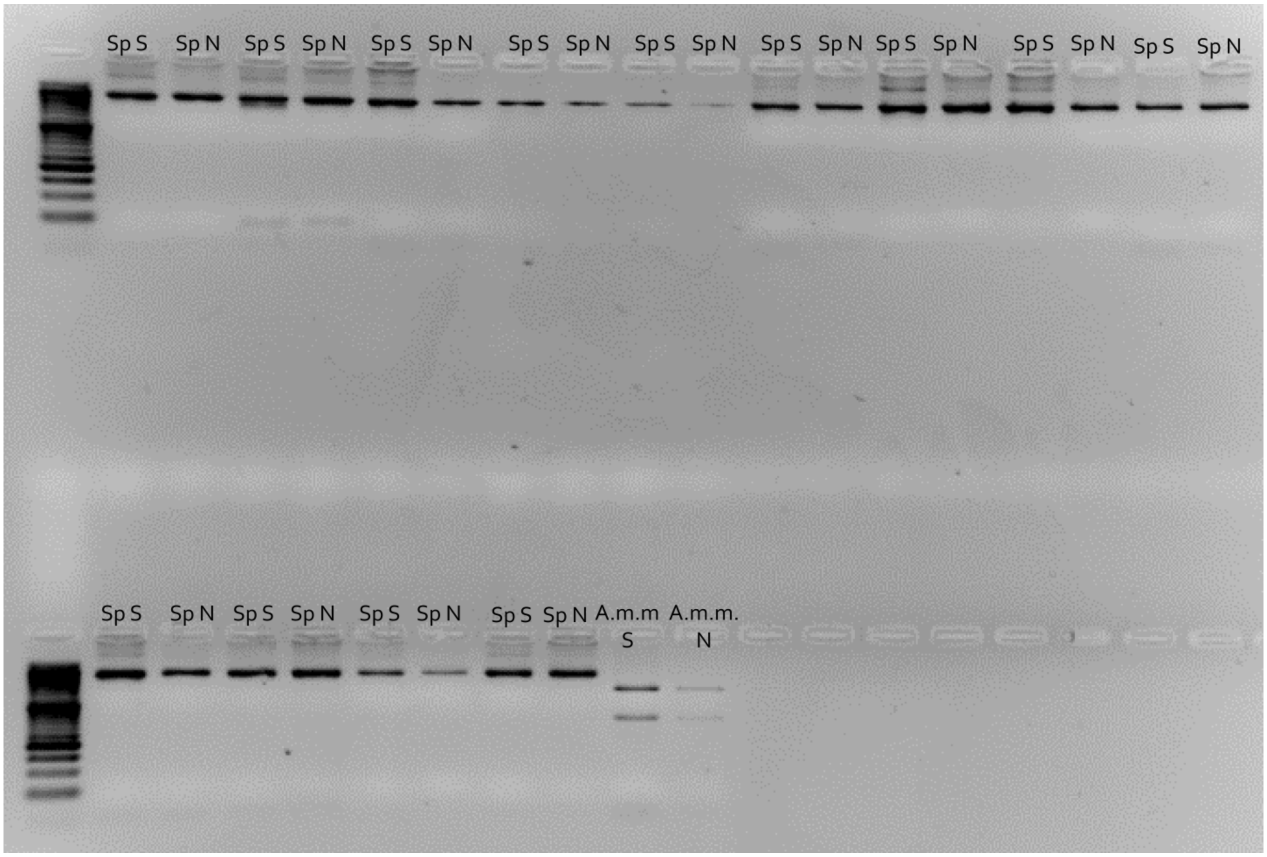

Figure S1. PCR-RFLP analysis of the COI-COII fragment digested with *Sty*I (S) and *Nco*I (N) enzymes, Sp – sample, A.m.m. – positive control of *Apis mellifera macedonica* digested with the same set of enzymes. The *A. m. macedonica* samples were provided by the Leonidas Haristos from the Division of Apiculture, Hellenic Agricultural Organization 'Demeter'.

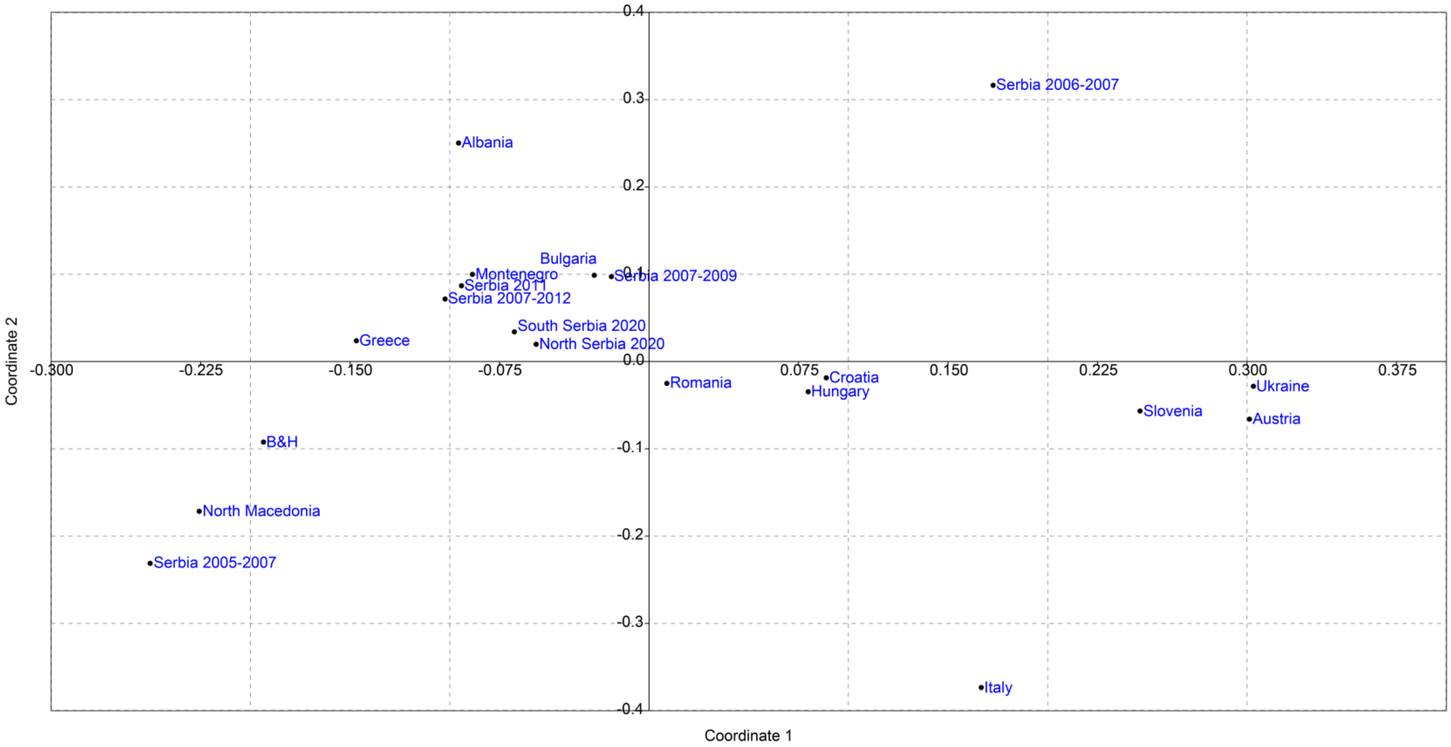

Figure S2. Non-metric multidimensional scaling plot of F_{ST} pairwise distances between the *A. mellifera* population of Serbia, two regions (North and South) and historical data from different time periods (2005-2007, 2006-2007, 2007-2012, 2011), and other *A. mellifera* populations based on the analysis of the variability of *tRNA*^{leu}*-cox2* intergenic region. The goodness of fit is expressed with the stress value which is 0.1567 for this data set. Population pairwise F_{ST} values are presented in Table S5.

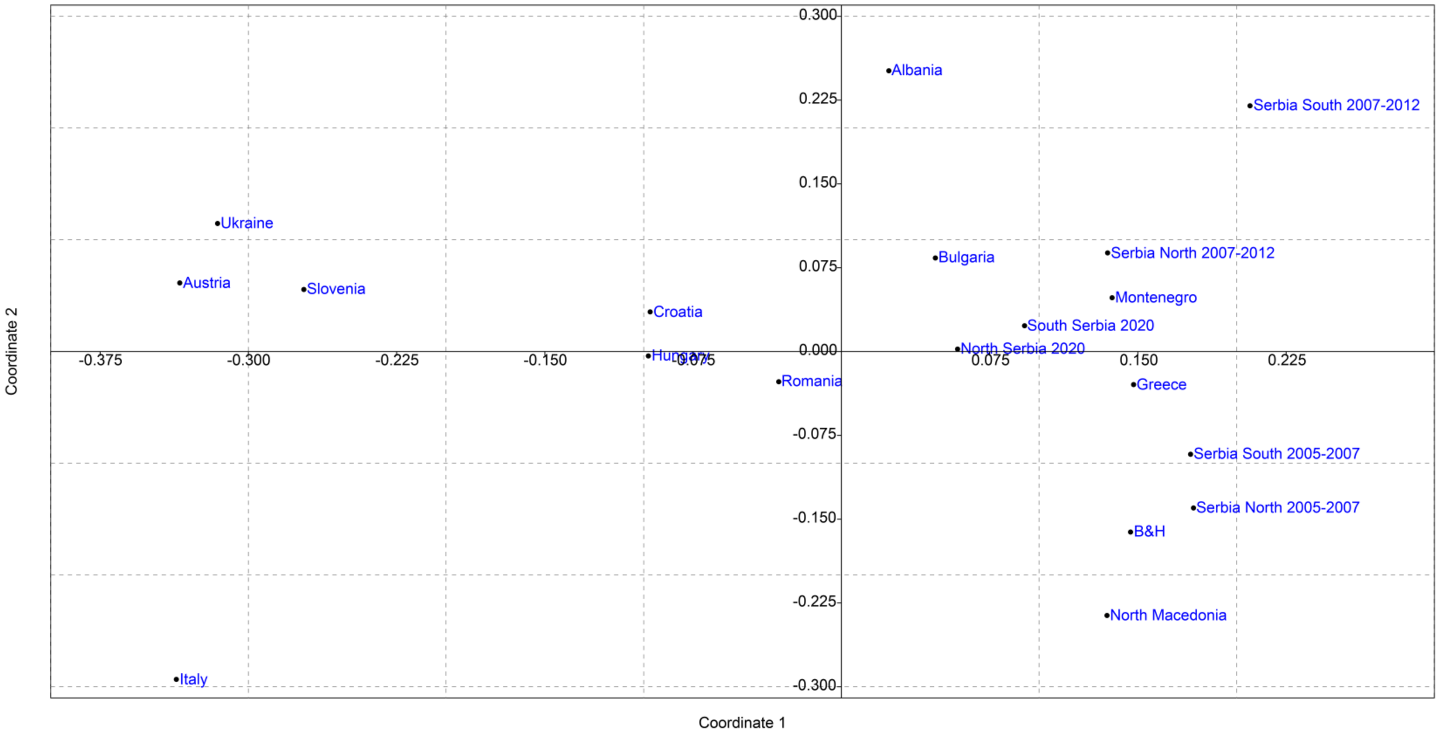

Figure S3. Non-metric multidimensional scaling plot of F_{ST} pairwise distances between the *A. mellifera* population of Serbia, two regions (North and South) and historical data from different time periods for the region of North and South Serbia (2005-2007, 2007-2012), and other *A. mellifera* populations based on the analysis of the variability of *tRNA^{leu}-cox2* intergenic region. The goodness of fit is expressed with the stress value which is 0.1395 for this data set. Population pairwise F_{ST} values are presented in Table S5.

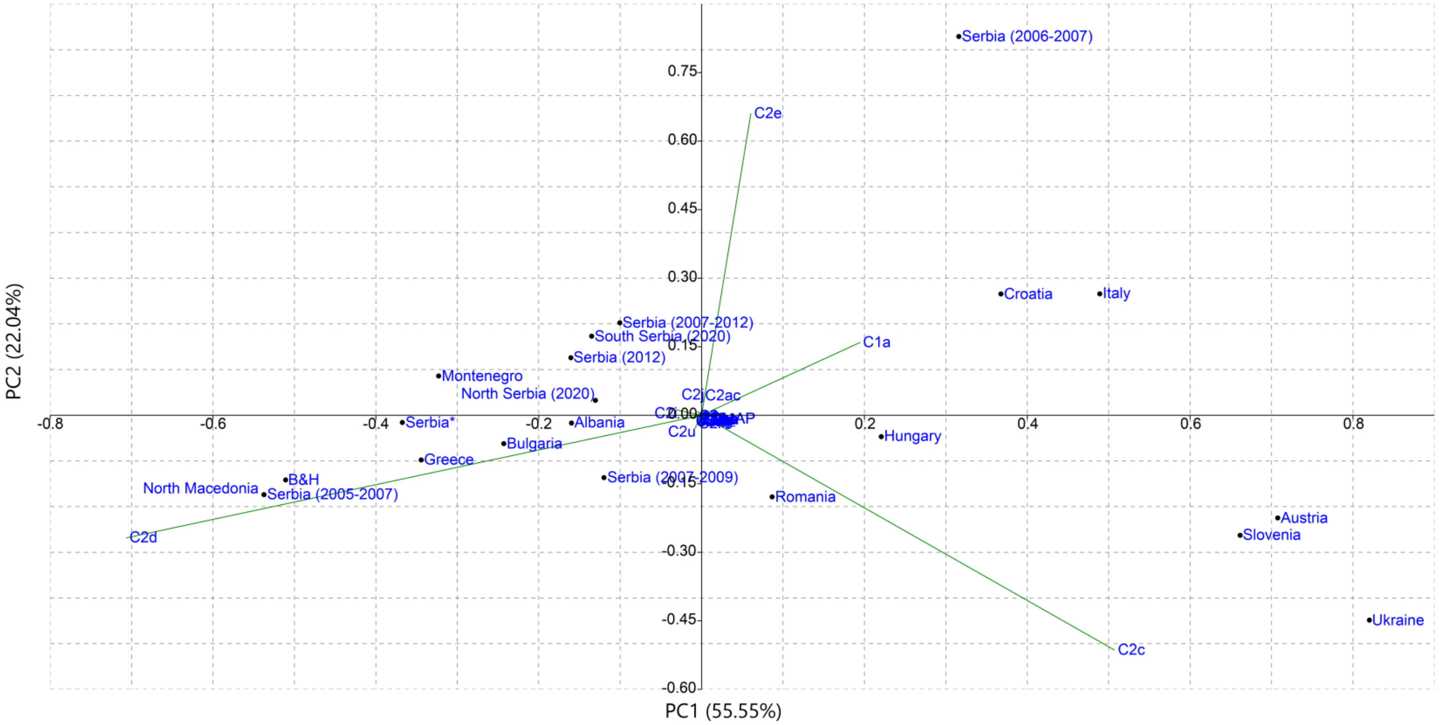

Figure S4. PC analysis based on mtDNA haplogroup frequencies in different *A. mellifera* populations. Samples for Serbia are presented as historical data from different time periods (2005-2007, 2006-2007, 2007-2012, 2011). The contribution of each haplogroup to the PCs is shown with green lines. Frequencies of mtDNA haplogroups and references are listed in Table S2 and S6.

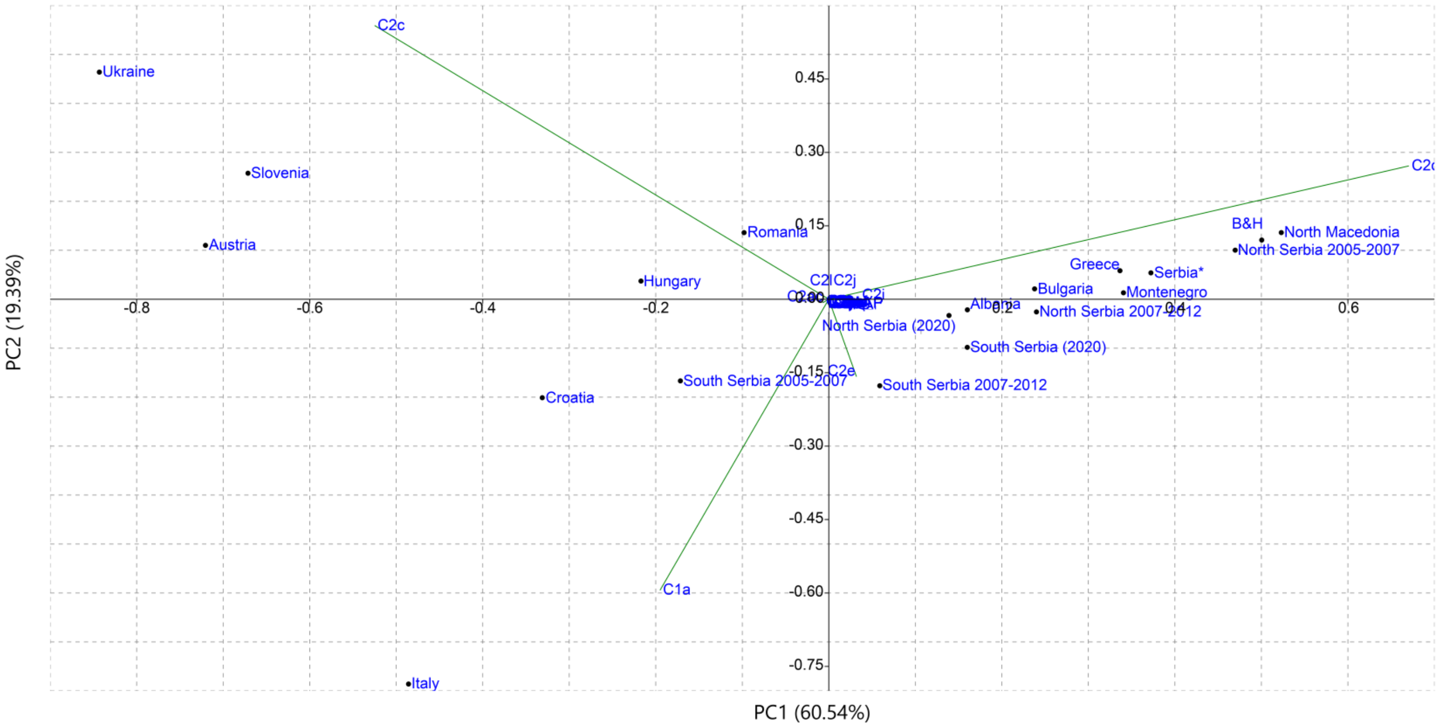

Figure S5. PC analysis based on mtDNA haplogroup frequencies in different *A. mellifera* populations. Samples for Serbia are presented as historical data from different time periods and regions of North and South Serbia (2005-2007, 2007-2012). The contribution of each haplogroup to the PCs is shown with green lines. Frequencies of mtDNA haplogroups and references are listed in Table S2 and S6-S7.
